# Prevalence of hypovitaminosis D and associated factors in obese Spanish children

**DOI:** 10.1038/nutd.2016.50

**Published:** 2017-03-13

**Authors:** T Durá-Travé, F Gallinas-Victoriano, M J Chueca-Guindulain, S Berrade-Zubiri

**Affiliations:** 1 Department of Pediatrics, School of Medicine, University of Navarra, Navarra Hospital Complex, Pamplona, Spain; 2 Department of Pediatrics, Navarra Hospital Complex, Pamplona, Spain; 3 Instituto de Investigación Sanitaria de Navarra (IdisNA), Pamplona, Spain

## Abstract

**Background/Objectives:**

Vitamin D deficiency may contribute to endocrine health and disease (diabetes, autoimmune thyroid diseases, polycystic ovarian syndrome, etc.). The aim of this study was to determine the prevalence and specific factors for hypovitaminosis D among children stratified by body mass index (BMI) in Northern Spain.

**Subjects/Methods:**

A cross-sectional clinical (sex, age, season of study visit, place of residence and BMI) and blood testing (calcium, phosphorous, calcidiol and parathyroid hormone (PTH)) were accomplished in 546 Caucasian individuals (aged 3.2–15.8 years). The BMI (*Z*-score) allowed establishing four groups: normal, overweight, obesity and severe obesity. The criteria of the *US Endocrine Society* were used for the definition of hypovitaminosis D.

**Results:**

Calcidiol levels were significantly higher in normal and overweight groups (*P*=0.001), whereas PTH levels were significantly higher in obesity and severe obesity groups (*P*=0.001). Hypovitaminosis D prevalence was significantly higher in severe obesity (81.1%) and obesity (68.2%) groups, whereas was lowest in overweight (55%) and normal (58.1%) groups (*P*=0.001). There was a negative correlation between calcidiol and PTH levels (*P*<0.01). Female (90.9%), adolescent group (88,2%), winter (100%) and autumn (82.4%) time and urban residence (94.1%) imply a higher prevalence of hypovitaminosis D in subjects with severe obesity (*P*<0.001). Female, puberal age, autumn, winter and spring time, urban residence and severe obesity were found to be independent predictors for hypovitaminosis D.

**Conclusions:**

Severe obesity could be considered as an associated factor for vitamin D deficiency, and, owing to its high prevalence, the implementation of systematic screening and hypovitaminosis treatment programs would be particularly useful.

## Introduction

Vitamin D and parathyroid hormone (PTH) are well known because of their role in bone metabolism and calcium homeostasis. Vitamin D deficiency leads to less absorption of dietary calcium and increased PTH secretion to maintain normal levels of serum calcium. Vitamin D deficiency induces osteoclastic activity and contributes to loss of bone mineral density.^1, 2^

Several observational studies have suggested that vitamin D deficiency may disrupt endocrine homeostasis in diabetes, autoimmune thyroid diseases and polycystic ovarian syndrome among others. Additional studies are needed to evaluate the underlying mechanisms.^2, 3, 4, 5, 6, 7, 8, 9^

Gender, age, race, season of the year in which serum is collected, sun exposure and childhood obesity have been associated with lower levels of serum calcidiol.^3, 5, 10, 11, 12, 13, 14, 15^ Vitamin D deficiency in obese individuals is attributed to several factors, such as decreased exposure to sunlight in obese subjects because of sedentary lifestyle, or to excessive vitamin sequestration within adipose tissue.^2, 3, 11, 16^ Fractures, tibia vara and slipped capital femoral epiphysis are more common in obese children perhaps because of decreased bone density in a setting of vitamin D deficiency.^17^ Thus, data on prevalence of vitamin D deficiency among children, stratified by body mass index (BMI) categories (normal weight, overweight, obesity and severe obesity), may inform about the need for screening and treatment.

The aim of this study is to determine the prevalence and specific factors (sex, age group, season of the year in which blood sample was taken and place of residence) for vitamin D deficiency among children stratified by BMI in Northern Spain.

## Materials and methods

### Patients

We examined a cross-sectional study of 546 individuals (237 males and 309 females) aged 3.28 to 15.85 years who underwent a clinical examination and blood testing in the Pediatric Endocrinology Unit in the period January 2014–December 2014. Pubertal stage was determined in each patient according to Tanner's criteria, and patients were classified in two different groups: school group (Tanner stage I) and adolescent group (Tanner stages II–V). Place of residence was categorized as urban or rural (> or <10 000 inhabitants, respectively).

The individuals were healthy white children in Navarra, Spain. They came from external consultations of the different pediatric subspecialties and no chronic pathologies that might affect growth, body composition, food ingestion or physical activity were detected previously. All patients who had received any medication (antiepileptic drugs or glucocorticoids) and vitamin D or calcium supplements were excluded.

### Clinical examination

Weight and height measurements were made in underwear while barefoot. Weight was measured using an Año-Sayol scale (reading interval 0 to 120 kg and a precision of 100 g), and height was measured using a Holtain wall stadiometer (reading interval 60 to 210 cm, precision 0.1 cm). The *Z*-score values for the BMI were calculated using the epidemiologic data contained whithin the program *Aplicación Nutricional*, from the Spanish Society of pediatric gastroenterology, hepatology and nutrition (Sociedad Española de Gastroenterología, Hepatología y Nutrición Pediátrica, available at http://www.gastroinf.es/nutritional/). The graphics from Ferrández *et al.* (Centro Andrea Prader, Zaragoza 2002) used as reference charts were included in (http://www.gastroinf.es/nutritional/).

The *Z*-score value for BMI allowed establishing the following groups:

- Normal: *Z*-score between −1.0 (15th percentile) and +1.0 (85th percentile).
- Overweight: *Z*-score >1.0 (85th percentile).
- Obesity: *Z*-score >2.0 (97th percentile).
- Severe obesity: *Z*-score >3.0 (99th percentile).

### Blood testing

Calcium, phosphorous and alkaline phosphatase plasma levels were measured during fasting by colorimetric methods using a COBAS 8000 analyzer (Roche Diagnostic, Mannheim, Germany). Intra- and interassay coefficients of variation were <5%.

Calcidiol was determined by a high-specific chemiluminiscence-immunassay (LIAISON Assay, Diasorin, Dietzenbach, Germany) with intra- and interassay coefficient of variation of 4.2–9.5% and 7.6–2.1%, respectively, and functional sensitivity of 4.0 ng ml^−1^. PTH was determined by a highly specific solid-phase, two-site chemiluminescent enzyme-labeled immunometric assay using an Immulite analyzer (DPC Biermann, Bad Nauheim, Germany) with intra- and interassay coefficient of variation of 3.8–6.9% and 3.1–7.2%, respectively, and functional sensitivity of 5.0 pg ml^−1^.^18^

Vitamin D deficiency was defined as calcidiol lower than 20 ng ml^−1^ (<50 nmol l^−1^). Vitamin D insufficiency is when calcidiol levels fluctuate between 20 and 29 ng ml^−1^ (50–75 nmol l^−1^) and vitamin D sufficiency is when calcidiol levels reach or overtake 30 ng ml^−1^ (>75 nmol l^−1^).^19, 20^ Secondary hyperparathyroidism was defined when PTH serum levels exceed 65 pg ml^−1^.^12, 21^

### Statistical analysis

Results are displayed as percentages (%) and means (M) with corresponding standard deviations (s.d.) and confidence intervals (95% CI). The statistical analysis (descriptive statistics, Student's *t*-test, analysis of variance, *χ*^2^ test, Pearson's correlation and multiple logistic regression analysis) was performed using the program Statistical Packages for the Social Sciences version 20.0 (Chicago, IL, USA). Statistical significance was assumed when *P*-value was <0.05.

Parents and/or legal guardians were informed and provided verbal consent for the participation in this study in all cases. This study was approved by the Ethics Committee for Human Investigation at our institution (in accordance with the ethical standards laid down in the 1964 Declaration of Hensinki and later amendments).

## Results

The BMI distribution of the enrolled participants and/or the presumed risk factors for hypovitaminosis D are summarized in Table 1. There were no significant differences between the distribution in relation to sex, season of blood sample and place of residence. However, the percentage of individuals suffering from obesity/severe obesity in the adolescent group was significantly higher with respect to the school group (*P*=0.003).

Table 2 shows and compares the mean values for the clinical characteristics and biochemical determinations according to BMI status. Calcidiol levels were significantly higher in normal and overweight groups (*P*=0.001), whereas the mean values for PTH levels were significantly higher in obesity and severe obesity groups (*P*=0.001). There were no significant differences in calcium, phosphate and alkaline phosphatase levels among the different groups according to BMI status.

**Figure 1:**
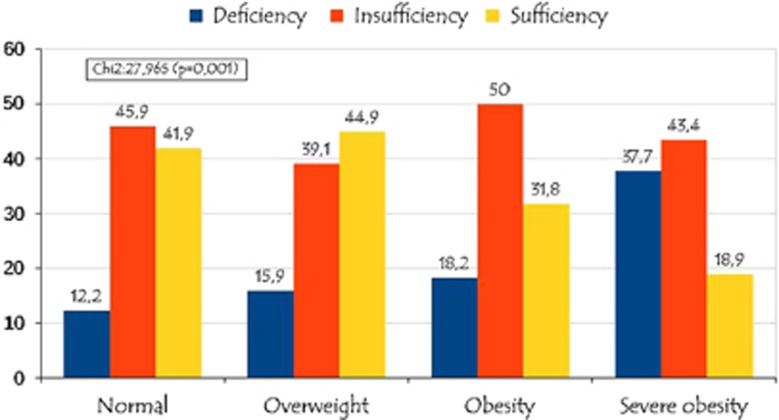
Prevalence of hypovitaminosis D in relation to the BMI status.

Figure 1 depicts and compares the prevalence of hypovitaminosis D in relation to the BMI status. Hypovitaminosis D prevalence (insufficiency and deficiency) was significantly higher in severe obesity (81.1%) and obesity (68.2%) groups, whereas the prevalence of hypovitaminosis D was lowest in overweight (55%) and normal (58.1%) groups (*P*=0.001). The frequency of hyperparathyroidism was significantly higher in severe obesity (26.1%) and obesity (9.1%) groups, whereas the prevalence of hyperparathyroidism was lowest in overweight (6.1%) and normal (2.5%) groups (*P*=0.001).

There was a negative correlation (*P*<0.01) between calcidiol and PTH levels (*r*=−0.245). In addition, there was also positive correlation (*p*<0.01) between PTH and BMI (*Z*-score) (*r*=0.268) and negative correlation (*P*<0.01) between calcidiol and BMI (*Z*-score) (*r*=−0.198).

Females in a normal BMI status show a prevalence of sufficiency and deficiency in vitamin D of 37.7% and 11.5%, respectively, and those females in a condition of severe obesity present with a prevalence of sufficiency and deficiency of 9.1% and 39.4%, respectively (*P*=0.001). There were no significant differences among males (*P*=0.079). Within the adolescent group, those in a normal BMI status showed a prevalence of sufficiency and deficiency of 34.1% and 15.9%, respectively, and those with severe obesity showed 11.8% and 44.1%, respectively (*P*=0.004). There were no significant differences within the school group (*P*=0.338). In winter time, prevalence of sufficiency and deficiency for individuals with normal BMI status was 44.6% and 19.6%, respectively, whereas the prevalence for those with severe obesity was 0% and 47.1%, respectively (*P*=0.006). In the summer time, the prevalence of sufficiency and deficiency for individuals in a normal BMI status was 84.4% and 4.4%, respectively, whereas the prevalence for those individuals with severe obesity was 50% and 20%, respectively (*P*=0.047). In autumn, the prevalence of sufficiency and deficiency for individuals in normal BMI status was 35.6% and 7.6%, respectively, whereas the prevalence in individuals with severe obesity was 17.6% and 52.9%, respectively (*P*=0.001). There were no significant differences in spring time (*P*=0.821). Within the group of urban residence, the prevalence of sufficiency and deficiency in vitamin D for individuals in a normal BMI status was 38.2% and 14.5%, respectively, whereas the prevalence for those individuals suffering from severe obesity was 5.9% and 47.1%, respectively (*P*=0.001). There were no significant differences in the group of rural residence (*P*=0.238).

The multiple logistic regression analysis for the presumed predictors of vitamin D status is represented in Table 3. Female gender, adolescent age, season of blood sample taken (autumn, winter and spring), urban residence and severe obesity were associated with an increased risk of vitamin D insufficiency. Furthermore, adolescent age, season of blood sample was taken (autumn and winter), urban residence and severe obesity were associated with an increased risk of vitamin D deficiency.

## Discussion

Sex, age, season of the year and place of residence have been described as independent factors as it was associated with hypovitaminosis D,^3, 5, 10, 11, 12, 13, 14, 15^ as indicated by the results of logistic analysis in this study; however, in this case, the analysis did not detect significant differences in the distribution of nutritional status in relation to this factors, except for a different proportion of individuals with obesity/severe obesity in the adolescent group, which was slightly higher. On the other hand, owing to the importance of geographical location and weather conditions in the cutaneous synthesis of vitamin D,^3, 12, 21, 22, 23, 24, 25^ it should be emphasized that Navarre is a Spanish region located in the north of the Iberian peninsula and characterized by a high frequency of precipitations and/or cloudiness and high latitude (42° north latitude), and these features could condition the levels of serum calcidiol.

Individuals with severe obesity showed relatively lower mean levels of calcidiol when compared with other BMI status (normal, overweight and obesity). The results display mean values for calcidiol decreasing substantively as BMI (*Z*-score) increases: from mean values of 28.2 ng ml^−1^ in a normal nutritional situation to 23.1 ng ml^−1^ in patients with severe obesity. In addition, a negative association between calcidiol and BMI (*Z*-score) is observed, as reported previously.^3, 5, 10, 13^ On the other hand, individuals with obesity and severe obesity exhibit mean PTH values significantly higher than individuals in normal BMI status and/or overweight. In fact, mean values for PTH substantively increase as BMI (*Z*-score) rises: from mean values of 31.1 pg ml^−1^ in those individuals with normal BMI status to 45.2 pg ml^−1^ in those individuals with severe obesity; therefore, there appears to be a positive association between PTH and BMI (*Z*-score). This means, a clear tendency to show low calcidiol levels and increased PTH levels in severe obesity in relation to other nutritional situations is detected; in this way, other authors have highlighted that this condition might constitute a metabolic and/or cardiovascular risk factor.^2, 4, 5, 6, 10, 13, 14, 16, 26, 27^

This reduction in the levels of serum calcidiol coinciding with increased adiposity is assumed to be due to enhanced sequestration of vitamin D in fat, a factor leading to decreased bioavailability.^2, 3, 11, 16, 28^ Consequently, lower serum calcidiol in obesity stimulates a rise in PTH, which in turn stimulates the renal hydroxylation of calcitriol and this condition elevates calcium influx into adipocytes. Intracellular calcium promotes lipogenesis and potentially reduces catecholamine-induced lipolysis.^16^ In fact, PTH has been postulated as an independent predictor of obesity; however, this hypothesis has been considered highly controversial, as weight loss in these individuals is associated with a normalization of vitamin D and PTH serum levels; that means, it rather seems as a consequence, not a cause, of excess body weight.^10^

Authors do not generally distinguish between obesity and severe obesity, as we have done in this work, and this detail could be of practical concern, mainly if we take into account that the logistic regression analysis determined how hypovitaminosis D status, both insufficiency and deficiency, was significantly associated with severe obesity.

In general, calcidiol concentrations below 20 ng ml^−1^ are considered to indicate vitamin D deficiency, whereas levels between 20 and 30 ng ml^−1^ indicate a relative insufficiency, and levels of 30 ng ml^−1^ or greater indicate sufficient vitamin D.^19, 20^ In this cross-sectional study, 56.8% of subjects with normal BMI status had suboptimal calcidiol levels (<30 ng ml^−1^) and 11.8% were vitamin D deficient (defined as calcidiol <20 ng ml^−1^). In contrast, the percentage of hypovitaminosis in individuals with severe obesity was 81.1% (insufficiency: 43.4% and deficiency: 37.7%). This means, individuals with severe obesity show a higher trend to present with vitamin D deficiency in comparison with other BMI status (normal, overweight and obesity), probably in relation to a more sedentary lifestyle and, consequently, a decreased sun exposure, and also to limited bioavailability of vitamin D caused by trapping in adipose tissue. These results, even when considered as high rates of hypovitaminosis D, are relatively moderate rates with respect to other published studies;^3, 11, 13, 14, 21, 29, 30, 31^ this fact may be due to the white race of such individuals, as, as it is well known, the difference in skin pigmentation in different ethnic groups implies a higher risk of hypovitaminosis D.^11, 12, 21, 32^

The analysis of the different factors associated to hypovitaminosis D (sex, age group, season of the year and place of residence) in relation to the different BMI status nutritional situations confirms that individuals with severe obesity show a higher tendency to vitamin D deficiency in females as well as in puberty, autumn and winter time and residence in an urban area with respect to the other BMI status. Nevertheless, the location of the residence in rural areas was not associated with a higher risk of suffering from vitamin D deficiency among the different BMI status. This finding would support the hypothesis that lifestyles (active outdoor life and sun exposure in rural areas) would be determining in the explanation of the differences among calcidiol serum levels and/or the prevalence of hypovitaminosis D.

Our study has several limitations, such as its cross-sectional nature and the absence of data about exercise, sun exposure and use of sunscreens. The difficulties in obtaining an adequate and accurate data collection deterred us from accomplishing the registration. A nutritional survey (dietary vitamin D intake, daily vitamin D supplementation, etc.) was not included. Our experience indicates that dairy product consumption in our environment is below the recommended amount, and fish consumption is very low among pediatric population,^33^ and only the dietary supplementation of vitamin D could condition the results obtained, but this is not an extended practice in our society.

In conclusion, severe obesity could be considered as a factor associated with vitamin D deficiency, and, owing to its high prevalence, the implementation of systematic screening and hypovitaminosis treatment programs would be particularly useful.

## Figures and Tables

**Table 1:** Demographics of the study participants stratified by BMI status

*Items*	*Normal,* n *(%)*	*Overweight,* n *(%)*	*Obesity,* n *(%)*	*Severe obesity,* n *(%)*	χ*^2^,* P-*value*
*Sex*					
Male	163 (68.8)	28 (11.8)	26 (11.0)	20 (8.4)	0.429
Female	192 (62.1)	41 (13.3)	42 (13.6)	34 (11.0)	
					
*Age group*					
Childhood	190 (72.2)	31 (11.8)	23 (8.7)	19 (7.2)	0.003
Adolescent	165 (58.3)	38 (13.4)	45 (15.9)	35 (12.4)	
					
*Season of study visit*					
Summer	46 (67.5)	8 (11.8)	4 (5.9)	10 (14.7)	0.625
Autumn	118 (65.2)	26 (14.4)	20 (11.0)	17 (9.44)	
Winter	112 (64.4)	21 (12.1)	24 (13.8)	17 (9.8)	
Spring	79 (64.2)	14 (11.4)	20 (16.0)	10 (8.1)	
					
*Residence*					
Rural	124 (65.3)	27 (14.2)	20 (10.5)	19 (10.0)	0.694
Urban	230 (64.8)	42 (11.8)	38 (13.5)	35 (9.9)	
Total	355 (65)	69 (12.6)	68 (12.5)	54 (9.9)	

Abbreviation: BMI, body mass index.

**Table 2:** Clinical and biochemical characteristics according to the BMI status (mean±s.d.)

*Items*	*Normal weight (*n=*355)*	*Overweight (*n=*69)*	*Obesity (*n=*68)*	*Severe obesity (*n=*54)*	*P-value*
Age (years)	9.65+3.34	10.43+2.6	11.09+2.4	11.11+3.28	0.001
BMI (*Z*-score)	−0.19±0.67	1.37+0.29	2.46+0.28	4.65+2.24	0.001
Calcium (mg dl^−1^)	9.98+0.35	10.01+0.37	10.05+0.35	9.96+0.36	0.610
Phosphorus (mg dl^−1^)	4.58+0.57	4.62+0.55	4.65+0.62	4.53+0.71	0.842
ALP (IU l^−1^)	234.92+81.36	243.86+82.41	253.21+91.16	220.26+99.71	0.253
PTH (pg ml^−1^)	31.09+15.94	33.12+16.25	39.89+20.56	45.22+21.99	0.001
Calcidiol (ng ml^−1^)	28.09+7.68	27.65+7.34	26.18+7.00	23.09+8.24	0.001

Abbreviations: ALP, alkaline phosphatase; BMI, body mass index; PTH, parathyroid hormone.

**Table 3:** Multiple logistic regression analysis for presumed risk factors for hypovitaminosis D

*Characteristics*	*Deficiency*		*Insufficiency*	
	*OR (95% CI)*	P*-value*	*OR (95% CI)*	P*-value*
*Sex*				
Male	Referent		Referent	
Female	1.07 (0.75–1.76)	0.788	1.66 (1.13.2.3)	0.009
				
*Age group*				
Children	Referent		Referent	
Adolescents	1.91 (1.14–3.19)	0.013	1.93 (1.31–2.48)	0.001
				
*Season of study visit*				
Summer	Referent		Referent	
Autumn	3.54 (1.25–10.9)	0.027	9.33 (4.57–19.03)	0.001
Winter	4.81 (1.59–14.87)	0.006	8.5 (4.15–17.41)	0.001
Spring	2.35 (0.71–7.71)	0.158	12.55 (5.85–26.95)	0.001
				
*Residence*				
Rural	Referent		Referent	
Urban	2.35 (1.22–3.85)	0.020	1.73 (1.49–2.09)	0.01
				
*BMI*				
Normal weight	Referent		Referent	
Overweight	1.34 (0.63–2.8)	0.438	0.77 (0.44–1.36)	0.476
Obesity	1.27 (0.61–2.64)	0.514	1.13 (0.62–2.07)	0.498
Severe obesity	4.32 (2.15–8.68)	0.001	4.23 (1.81–9.89)	0.001

Abbreviations: BMI, body mass index; CI, confidence interval; OR, odds ratio.
